# Treatment of Patients With De Novo Small-Vessel Coronary Lesions: Analysis of Six Randomised Controlled Trials Comparing Paclitaxel-Coated Balloons With Drug-Eluting Stents

**DOI:** 10.7759/cureus.69983

**Published:** 2024-09-23

**Authors:** Andrea Messori, Sabrina Trippoli

**Affiliations:** 1 Health Technology Assessment Unit, Regione Toscana, Florence, ITA; 2 Scientific Committee, Società Italiana di Farmacia Clinica e Terapia, Turin, ITA

**Keywords:** small-vessel coronary lesions, balloon angioplasty, sirolimus-coated ballons, paclitaxel-coated balloons, meta-analysis, coronary stents

## Abstract

Both paclitaxel-coated balloons (PCB) and drug-eluting stents (DES) are indicated for the treatment of de novo small-vessel coronary lesions. Since the evidence comparing these two types of devices is limited, we undertook a meta-analysis on this issue.

Our meta-analysis compared the efficacy of PCB vs. DES in the treatment of patients with de novo coronary lesions of size ≤ 2.75 mm. Only randomized controlled trials (RCTs) were included. The two treatments under comparison were PCB vs DES; the endpoint was the rate of major adverse cardiovascular events (MACE). Our statistical methods were based on the reconstruction of individual patient data from Kaplan-Meier curves using the IPDfromKM algorithm. After this reconstruction, our statistical calculations included hazard ratio (HR) estimation with a 95% confidence interval (CI), assessment of between-trial heterogeneity, and risk of bias for each RCT.

Our literature search identified six RCTs that met our inclusion criteria (PICCOLETO, BELLO, RESTORE SVD, BASKET-SMALL2, PICCOLETO-II, and DISSOLVE). In our main analysis, the six treatment groups using PCB were compared with the six control groups using DES. The results showed an HR of 1.029 (95%CI, 0.7446 to 1.422; P=0.86) over a follow-up of 36 months. Heterogeneity analysis across the six control groups showed worse outcomes in the BELLO trial and better outcomes in the three trials employing a limus-eluting stent. To evaluate trial heterogeneity through the comparison of the six PCB arms, five trials showed similar outcomes while the BELLO trial fared significantly worse. Risk of bias for each RCT was appropriate.

Our results indicate that in de novo small-vessel coronary lesions, PCB and DES showed similar outcomes, despite some cross-study variability. Our results provided meta-analytic confirmation that no recommendations can be made in favor of PCB or DES in the treatment of de novo small-vessel coronary lesions based on current data.

## Introduction and background

Drug-coated balloons are specialized coronary devices comprised of a semi-compliant balloon catheter with an engineered coating that allows the delivery of antiproliferative agents locally to the vessel wall during percutaneous coronary intervention. Although these devices were initially developed more than a decade ago [1], their potential in coronary interventions has recently attracted renewed interest. A recent review has emphasized the relevance of racial and socioeconomic determinants of this disease condition [2].

Originally designed to overcome the limitations of conventional balloon angioplasty and stenting, drug-coated balloons aim to improve the outcomes of DES without leaving a permanent implant. Currently, drug-coated balloons are mainly used for the treatment of in-stent restenosis after percutaneous transluminal angioplasty; less frequently, they are also used for the treatment of de novo coronary lesions in small-caliber vessels [1]. The literature on the potential benefits of drug-eluting balloons is relatively recent [1] but still limited; therefore, an updated meta-analysis on this clinical question may be of value. 

Several drug-coated balloons are available on the market, most of which use paclitaxel formulations (PCB); the other agents commercially available include sirolimus and, only in China, zotarolimus. Paclitaxel entered the European interventional cardiology market in 2007, while the first sirolimus-coated balloon (MagicTouch, Concept Medical, Tampa, FL) received its Conformité Européenne (CE) certification in 2016. However, it is still unclear whether sirolimus is an alternative to paclitaxel because it is generally agreed that the latter remains the drug of choice [1].

Paclitaxel is a cytotoxic drug, acting by binding irreversibly to microtubules (β subunit of tubulin) and exerting a persistent duration of action in vascular cells, whereas sirolimus and its equivalents are cytostatic in that they bind reversibly to FKBP-12, forming a complex with the mammalian target of rapamycin, arresting the cell cycle at the junction between G1 and S phases [1]. However, the cytotoxic properties of paclitaxel have been demonstrated only at tissue concentrations >100 ng/mg which are usually found in the first few hours after application. Otherwise, the drug exhibits cytostatic properties similar to those of sirolimus in lower tissue concentrations. Because of its lipophilic nature, paclitaxel remains in the arterial wall for longer periods, inhibiting smooth cell proliferation and leading to positive remodeling of the arterial wall [1].

For the treatment of de novo lesions in small caliber vessels, PCBs have been evaluated in fewer trials than the use of these balloons for in-stent restenosis. In fact, the evidence comparing drug-coated balloons with drug-eluting stents (DES) is based on a rather limited number of trials [1,3,4-9]. On the one hand, small-vessel coronary lesions are more difficult to treat; on the other hand, the paucity of controlled trials underscores the need for careful analysis of the available literature. In particular, the comparison of drug-eluting balloons with DES is important in this specific context. 

## Review

Methods

A standard literature search was conducted using the PubMed database. The selection of pertinent articles was made according to the PRISMA algorithm [10]. The search term used in the initial search was as follows: ("drug-eluting stent*" OR "paclitaxel-eluting stent*") AND ("drug-eluting balloon*" OR "paclitaxel-eluting balloon*") AND "small vessel". Only randomized controlled trials (RCTs) were included. The endpoint of our analysis was the rate of major adverse cardiovascular events (MACE). Other inclusion criteria were the comparison between a drug-coated balloon vs a DES in de novo small-vessel coronary lesions and the use of the above-mentioned endpoint. Besides the lack of a randomized design, other exclusion criteria were the absence of a Kaplan-Meier curve, the use of an endpoint other than MACE, and the duplicate publication of the same trial.

Our meta-analysis was based on the reconstruction of individual patient data using the artificial intelligence (AI) software IPDfromKM [11,12]. Briefly, the Kaplan-Meier curves published in the clinical trials are analyzed through an AI algorithm that generates a database of "reconstructed" patients; in this patient database, individual follow-up durations are included along with the status of each patient at the last date of the follow-up (with event or without event). The operational details of this method have previously been described in an article published in Cureus in 2021 [12]. 

After this reconstruction of individual patient data, all statistical calculations comparing PCB vs. DES were performed using the same standard time-to-event methods as those used in clinical trials. Accordingly, our statistical analysis used the Cox regression model to estimate the hazard ratio (HR) with a 95% confidence interval (CI). After pooling all patients treated with PCB and those treated with DES, two standard Kaplan-Meier curves based on "reconstructed" patients of the two treatment groups were generated and subjected to HR estimation. Heterogeneity in the six control groups was assessed using the Wald test and the likelihood ratio test. Finally, the risk of bias of each of the included RCTs was assessed according to Version 1 of the Cochrane risk of bias tool [13].

Results

Literature Search and Included Trials

**Figure 1:**
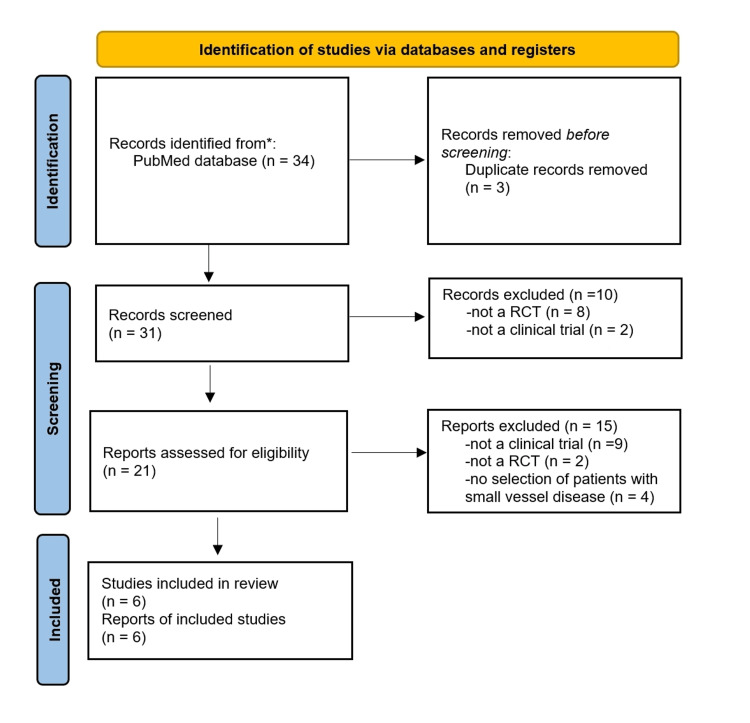
PRISMA algorithm. Abbeviations: RCT, randomized controlled trial.

Figure 1 shows the selection process of our literature search based on the PRISMA algorithm. Six RCTs comparing PCB with DES were identified.

Table 1 summarizes the main characteristics of these six trials, including the endpoint definitions and the incidence of the endpoint in each arm. There were six arms treated with PCB and six arms treated with DES (three eluting paclitaxel, two zotarolimus, and one everolimus). It should be noted that the trial by Liu et al. [9] investigated a balloon developed in China (DISSOLVE), which is still undergoing regulatory approval. 

**Table 1:** Main characteristics of the six RCTs included in our meta-analysis. *No censored cases were assumed during the nine months of follow-up. Abbreviations: PCB, paclitaxel-coated balloon; PES, paclitaxel-eluting stent; MACE, major adverse cardiovascular event.

Authors	Year of publication	Treatments and no. of patients		Total no. of patients	Endpoint	Follow-up duration (months)	Event rate (n/N)	
		Balloon group	Stent group				PCB	DES
Cortese et al., PICCOLETO study [4]	2010	PCB (Dior), N=28	Taxus PES, N=29	N=57	MACE	9	10*/28	4*/29
Naganuma et al., BELLO study [5]	2015	PCB (Impact Falcon), N=90	Taxus PES, N=92	N=182	MACE	24	13/90	23/92
Tang et al., RESTORE SVD [6]	2018	PCB (Restore), N=116	Resolute zotarolimus-eluting stent, N=114	N=230	MACE	12	9/116	4/114
Jeger et al., BASKET-SMALL2 study [7]	2020	PCB (Sequent Please), N=382	Xience everolimus-eluting stent (72%) or Taxus PES (28%), N=376	N=768	MACE	36	53/382	53/376
Cortese et al., PICCOLETO-II [8]	2023	PCB (Elutax SV), N=118	Xience everolimus-eluting stent, N=114	N=232	MACE	35	11/118	21/114
Liu et al., DISSOLVE [9]	2024	PCB (Dissolve), N=129	Resolute zotarolimus-eluting stent, N=118	N=247	MACE	12	27/129	16/118

Risk of Bias in the Six Included RCTs

**Figure 2:**
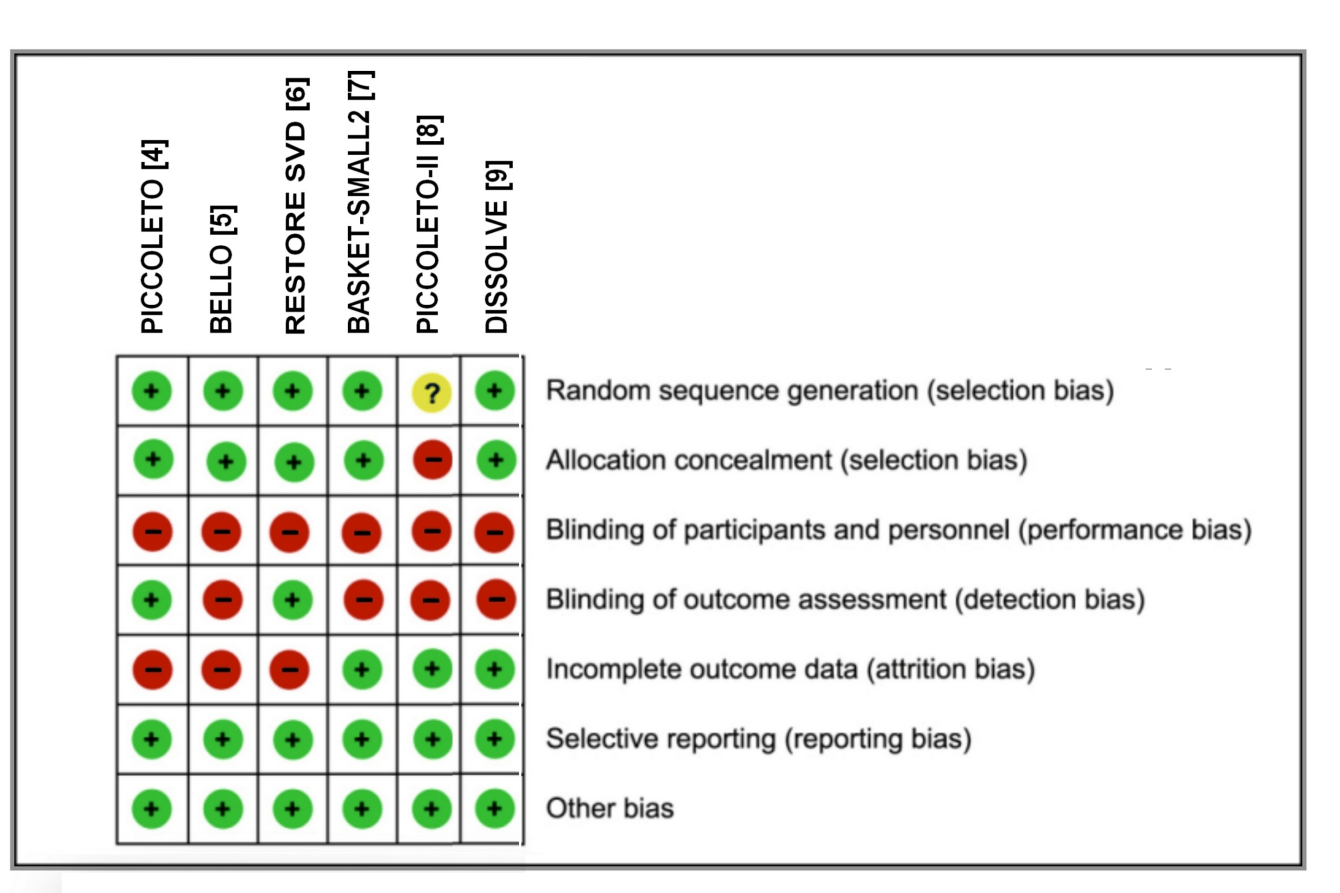
Risk of bias in the six included randomized controlled trials Assessed according to risk of bias tool (Version 1) of the Cochrane collaboration [13]. The three symbols ("+", "-", and "?") indicate a positive score, a negative score, and missing information, respectively.

Figure 2 shows the results of our assessment of the risk of bias for each RCT. Its results show that the quality of included RCTs was appropriate. 

Incidence of MACE in Included Trials: Meta-Analysis and Assessment of Heterogeneity

**Figure 3:**
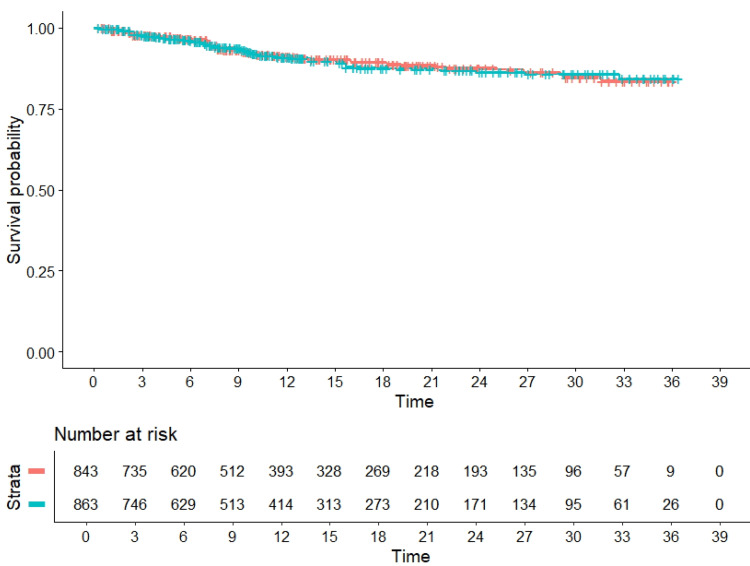
Results of the meta-analysis: incidence over time of major adverse cardiac events. Data from six randomized controlled trials (see Table 1). Time is measured in months; red, paclitaxel-coated balloons; and green, drug-eluting stents.

The results of our meta-analysis are shown in Figure 3. The main finding is represented by the pooled HR, which was 1.029 (95% CI, 0.7446 to 1.422; P=0.86); it can be seen that while the HR of the overall analysis was close to 1, its 95% CI was quite wide.

**Figure 4:**
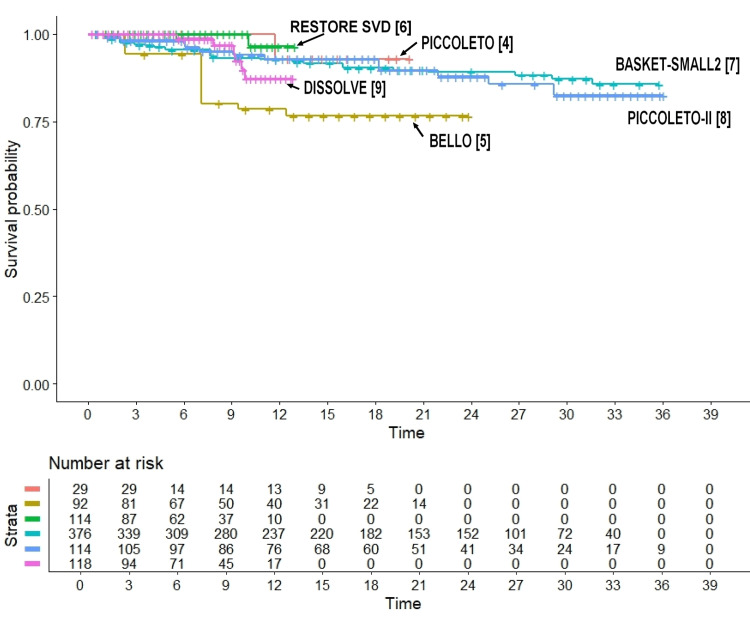
Cross-trial heterogeneity in the six control groups. The PICCOLETO trial [4] shows the worst outcome. Time is measured in months.

The cross-trial heterogeneity in the six control groups was significant (likelihood ratio test= 17.02 on 5 df, p=0.004; Wald test = 16.3 on 5 df, p=0.006), probably due to the presence of worse outcomes in the BELLO trial (Figure 4). The reason for these worse outcomes in the BELLO trial remains unclear. This heterogeneity prevented the demonstration of the non-inferiority of PCB versus DES. On the other hand, the four control groups (from the RESTORE SVD trial [6], the BASKET-SMALL2 trial [7], the PICCOLETO-II trial [8], and the DISSOLVE trial [9]) based on second-generation DES showed a better pattern of outcomes.

**Figure 5:**
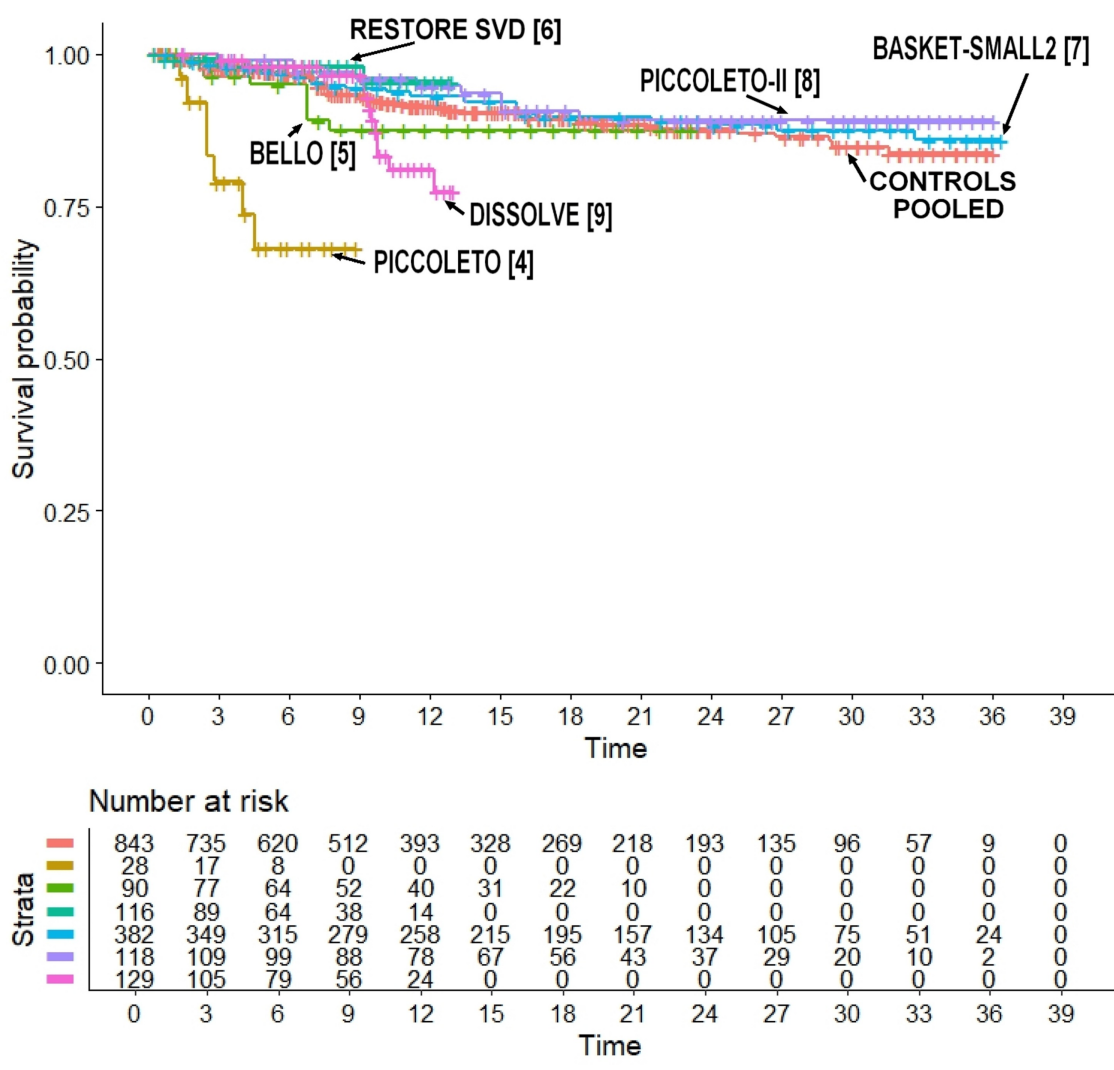
Comparison of each of the six treatments' arm vs the six control groups pooled together. The PICCOLETO trial [4] shows the worst outcome. Time is measured in months.

Finally, Figure 5 compares the Kaplan-Meier curves of each of the six PCB arms with the six control groups pooled together. These data clearly show that the treatment arm of the PICCOLETO trial [4] showed a markedly worse outcome compared with both the other five PCB arms and the six control arms pooled together.

Discussion

Our study provides original evidence on a controversial issue represented by the role of drug-coated balloons in the treatment of coronary lesions, particularly those of small vessels. In fact, there are three main categories of coronary lesions for which drug-coated balloons are indicated.

The first indication is the second-line treatment of in-stent restenosis after the failure of a DES as a first-line treatment. While the rate of in-stent restenosis has been remarkably reduced with second-generation DES, its incidence remains at 5% to 10% [3]. Until a few years ago, plain old balloon angioplasty (POBA) along with DES was the standard of care for the treatment of in-stent restenosis, but more recently, drug-coated balloons, such as PCB or limus-coated balloons, have become available as a technological advancement over POBA. However, while the evidence in favor of drug-coated balloons over POBA is based on five RCTs (according to Bhogal et al. [1]), more numerous RCTs are available for the comparison of drug-coated balloons vs. DES in this disease condition (10 RCTs according to Bhogal et al. [1]). However, only one of these RCTs (in which the Agent Drug-Coated Balloon device was used) has shown a clear superiority of balloons over DES; for this reason, only this device has received FDA approval for in-stent restenosis. Furthermore, one drawback in terms of clinical evidence is that the incremental benefit of drug-coated balloons over DES, based on recent data, has been evaluated only in the narrative review by Bhogal et al. [1], and no formal meta-analysis is available on this issue. Most RCTs in this area have tested PCBs, and there is only one RCT that has tested a sirolimus-coated balloon [1].

The second indication is the first-line treatment of large-vessel coronary lesions (size > 3.7 cm); this is an indication for which a heterogeneous number of trials with very different designs have been reported [14-17]. Most of these trials were based on a single-arm noncomparative design; four RCTs have been published on this disease condition, however, these failed to prove any advantage of drug-coated balloons vs DES.

The third indication is the de novo treatment of small-vessel coronary lesions, which is the specific subject of the present meta-analysis. Our results have clearly shown a very similar efficacy between PCBs and DES (eluting paclitaxel, everolimus, or zotarolimus) in this indication with respect to the rate of MACE, which is the most relevant endpoint (Figure 2). 

Limitations of the Study

The presence of a significant level of between-studies heterogeneity is the main limitation of our analysis. In fact, while four of the six included RCTs (namely: the RESTORE SVD trial [6], the BASKET-SMALL2 trial [7], the PICCOLETO-II trial [8], and the DISSOLVE trial [9]) used in the control arm a second-generation DES (eluting either zotarolimus or everolimus), the remaining two RCTs employed a first-generation DES (eluting paclitaxel). On the one hand, our overview of these trials simply reflects the currently available information on the effectiveness of the drug-coated balloons; on the other, it is interesting to note that in Figure 3, the three control arms treated with limus-eluting stents were those showing the highest efficacy.

Comparing our study with the evidence already published on this topic, the review by Bhogal et al. [1], which we have cited several times, is certainly the most important paper published to date on the use of drug-coated balloons in coronary lesions. As mentioned above, the study by Bhogal et al. [1], which includes a specific analysis of small-vessel lesions, is a narrative review. Two other narrative reviews on this topic are worth mentioning: the first was published by Arslani et al. [18] in 2021, and the second by Nestelberger et al. [19] in 2019; however, both are older than that of Bhogal et al. [1].

Comparison With Binary Meta-Analyses 

In the field of binary meta-analysis (which is a simpler tool compared to the IPDfromKM method), three publications on this topic can be found in the recent literature [3,19,20]: the first was published by Felbel et al. [3] in August 2023, the second by Murphy et al. [20] in April 2023, and the third by Wu et al. [21] in April 2021. Felbel et al. [3] included a total of 37 studies because both observational and randomized studies were eligible for their analysis; however, these authors reported a total of four RCTs comparing PCB vs. DES (in contrast to the six RCTs included in our IPDfromKM analysis). Similarly, the two meta-analyses by Murphy et al. [20] and Wu et al. [21] also included four RCTs.

From a methodological point of view, the main advantage of our analysis is the use of "reconstructed" patients generated according to the IPDfromKM method [11,12]; this method has the advantage of providing a more detailed representation of the results of individual trials and facilitates indirect comparisons. The main limitation of the IPDfromKM method is that it focuses mainly on the primary endpoint of the trials; secondary clinical endpoints can be evaluated provided that the original trial has reported a specific Kaplan-Meier plot based on the endpoint in question. Interestingly, the IPDfromKM method has been widely used in the last three years, especially in cardiology [22].

## Conclusions

In conclusion, our results showed that the outcomes of PCB and DES are essentially identical, despite some cross-trial variability. Therefore, our study provided meta-analytic confirmation that, based on the available evidence, no recommendation can be made in favor of either PCB or DES in the de novo treatment of small-vessel coronary lesions. Although three agents can be used in DES (paclitaxel, sirolimus, and zotarolimus), the clinical trials evaluating drug-coated balloons are more homogeneous because all RCTs used paclitaxel as a common agent.

## References

[1] Bhogal S, Hill AP, Merdler I, Wermers JP, Ben-Dor I, Waksman R (2024). Drug-coated balloons for coronary artery disease: An updated review with future perspectives. Cardiovasc Revasc Med.

[2] Borkowski P, Borkowska N, Mangeshkar S, Adal BH, Singh N (2024). Racial and socioeconomic determinants of cardiovascular health: a comprehensive review. Cureus.

[3] Felbel D, Bozic F, Mayer B (2023). Drug-coated balloon: an effective alternative to stent strategy in small-vessel coronary artery disease-a meta-analysis. Front Cardiovasc Med.

[4] Cortese B, Micheli A, Picchi A, Coppolaro A, Bandinelli L, Severi S, Limbruno U (2010). Paclitaxel-coated balloon versus drug-eluting stent during PCI of small coronary vessels, a prospective randomised clinical trial. The PICCOLETO study. Heart.

[5] Naganuma T, Latib A, Sgueglia GA (2015). A 2-year follow-up of a randomized multicenter study comparing a paclitaxel drug-eluting balloon with a paclitaxel-eluting stent in small coronary vessels the BELLO study. Int J Cardiol.

[6] Tang Y, Qiao S, Su X (2018). Drug-coated balloon versus drug-eluting stent for small-vessel disease: the RESTORE SVD China randomized trial. JACC Cardiovasc Interv.

[7] Jeger RV, Farah A, Ohlow MA (2020). Long-term efficacy and safety of drug-coated balloons versus drug-eluting stents for small coronary artery disease (BASKET-SMALL 2): 3-year follow-up of a randomised, non-inferiority trial. Lancet.

[8] Cortese B, Testa G, Rivero F, Erriquez A, Alfonso F (2023). Long-term outcome of drug-coated balloon vs drug-eluting stent for small coronary vessels: PICCOLETO-II 3-year follow-up. JACC Cardiovasc Interv.

[9] Liu S, Zhou Y, Shen Z (2024). Comparison of drug-coated balloon and drug-eluting stent for the treatment of small vessel disease (from the dissolve SVD randomized trial). Am J Cardiol.

[10] Page MJ, McKenzie JE, Bossuyt PM (2021). The PRISMA 2020 statement: an updated guideline for reporting systematic reviews. BMJ.

[11] Liu N, Zhou Y, Lee JJ (2021). IPDfromKM: reconstruct individual patient data from published Kaplan-Meier survival curves. BMC Med Res Methodol.

[12] Messori A (2021). Synthetizing published evidence on survival by reconstruction of patient-level data and generation of a multi-trial Kaplan-Meier curve. Cureus.

[13] Higgins JP, Green S (eds) (2011). Cochrane Handbook for Systematic Reviews of Interventions, version 5.1.0. https://handbook-5-1.cochrane.org/.

[14] Merinopoulos I, Gunawardena T, Wickramarachchi U (2021). Long-term safety of paclitaxel drug-coated balloon-only angioplasty for de novo coronary artery disease: the SPARTAN DCB study. Clin Res Cardiol.

[15] Vos NS, Dirksen MT, Vink MA (2014). Safety and feasibility of a PAclitaxel-eluting balloon angioplasty in Primary Percutaneous coronary intervention in Amsterdam (PAPPA): one-year clinical outcome of a pilot study. EuroIntervention.

[16] Scheller B, Ohlow MA, Ewen S (2020). Bare metal or drug-eluting stent versus drug-coated balloon in non-ST-elevation myocardial infarction: the randomised PEPCAD NSTEMI trial. EuroIntervention.

[17] Vos NS, Fagel ND, Amoroso G (2019). Paclitaxel-coated balloon angioplasty versus drug-eluting stent in acute myocardial infarction: the REVELATION randomized trial. JACC Cardiovasc Interv.

[18] Arslani K, Jeger R (2021). Drug-coated balloons for small coronary disease-a literature review. Curr Cardiol Rep.

[19] Nestelberger T, Jeger R (2019). Drug-coated balloons for small coronary vessel interventions: a literature review. Interv Cardiol.

[20] Murphy G, Naughton A, Durand R, Heron E, McCaughey C, Murphy RT, Pearson I (2023). Long-term outcomes for drug-eluting balloons versus drug-eluting stents in the treatment of small vessel coronary artery disease: a systematic review and meta-analysis. Interv Cardiol.

[21] Wu X, Li L, He L (2021). Drug-coated balloon versus drug-eluting stent in patients with small-vessel coronary artery disease: a meta-analysis of randomized controlled trials. Cardiol Res Pract.

[22] Messori A (2024). Reconstruction of individual-patient data from the analysis of Kaplan-Meier curves: the use of this method has extended from oncology to cardiology - List of 28 studies in cardiology [PREPRINT]. Open Science Framework.

